# Molecular Detection of Insecticide Resistance-Associated Mutations in *vgsc*, *ace-1*, and *rdl* Genes of *Anopheles albimanus* in Panama

**DOI:** 10.3390/insects16111115

**Published:** 2025-10-31

**Authors:** Chystrie A. Rigg, Andrés Cabrera, Vanessa Vásquez, Ana María Santamaría, Lorenzo Cáceres, Lisbeth A. Hurtado, Gonzalo Greif, José E. Calzada

**Affiliations:** 1 Departamento de Investigación en Parasitología, Instituto Conmemorativo Gorgas de Estudios de la Salud, Panama 0816-02593, Panama; chrigg@gorgas.gob.pa (C.A.R.); vvasquez@gorgas.gob.pa (V.V.); asantamaria@gorgas.gob.pa (A.M.S.); 2 Programa de Desarrollo de las Ciencias Básicas, Universidad de la República, Montevideo 11400, Uruguay; 3 Unidad Académica de Parasitología y Micología, Facultad de Medicina, Universidad de la República, Montevideo 11550, Uruguay; acabrera@higiene.edu.uy; 4 Unidad de Biología Molecular, Laboratorio de Interacciones Hospedero-Patógeno, Instituto Pasteur de Montevideo, Montevideo 11400, Uruguay; 5 Departamento de Entomología Médica, Instituto Conmemorativo Gorgas de Estudios de la Salud, Panama 0816-02593, Panama; lcaceres@gorgas.gob.pa; 6 Departamento de Análisis Epidemiológico y Bioestadísticas, Instituto Conmemorativo Gorgas de Estudios de la Salud, Panama 0816-02593, Panama; lhurtado@gorgas.gob.pa; 7 Facultad de Medicina Veterinaria, Universidad de Panamá, Panama 0816-03366, Panama

**Keywords:** *Anopheles albimanus*, insecticide-resistance, mutations, malaria, comarcas, Panama

## Abstract

This study evaluated the molecular resistance profile to insecticides and the genetic diversity of *Anopheles albimanus* populations from malaria endemic comarcas in Panama, a country in Mesoamerica aiming to eliminate local malaria transmission. Molecular screening was performed in 891 *An. albimanus*, distributed in 162 pools, and collected between 2011 and 2023. Pools were molecularly examined to detect natural infection with *Plasmodium* and sequenced to assess mutations in genes (*vgsc*, *ace-1* and *rdl*) associated with resistance to commonly used insecticides. A high molecular infection rate by *Plasmodium vivax* was detected in all comarcas throughout the study period, and *P. falciparum* infections were detected in the last two years (2022–2023) in the east region. Mutations associated with pyrethroids/DDT resistance (H973Y and L1014F/C) and to organophosphorus/carbamate resistance (G119S) were detected at high frequencies (50.8% and 70%, respectively) in eastern comarcas but were absent from comarcas located west of the Panama Canal. Mutations in the *rdl* gene, associated with resistance to cyclodienes and neonicotinoids, were also frequently present. *Anopheles* populations from the western side were highly homogenous, suggesting a clonal expansion, contrasting with eastern samples, which exhibited a high genetic diversity. Our study provides a valuable baseline for planning future molecular vector surveillance studies in the region. It also provides valuable information to the vector control program in Panama to guide insecticide selection for IRS.

## Simple Summary

This study evaluated mosquito populations in Panama to better understand how resistant they are to the insecticides used to control them, and how genetically diverse they are. The focus was on *Anopheles albimanus*, the main vector of malaria in the country. Nearly 900 mosquitoes (162 pools) were collected over a 12-year period (2011–2023) from indigenous areas (areas) with frequent malaria cases. Mosquitoes were checked for infections with malaria parasites and for genetic changes that might make the mosquitoes resistant to insecticides. The results showed that many mosquitoes carried malaria parasites, especially *Plasmodium vivax*, and that *Plasmodium falciparum* had also reappeared recently. Mutations associated with resistance against different classes of insecticides—such as pyrethroids, organophosphates, organochlorines (DDT and cyclodienes) and neonicotinoids—were found mostly in the eastern regions of Panama. In contrast, mosquito populations in the western region showed little genetic variation and few signs of resistance. These findings are important because they help public health officials choose the right insecticides to control mosquitoes and prevent malaria in Panama.

## 1. Introduction

Mesoamerica has been identified by the World Health Organization (WHO) as a region with high potential for malaria elimination due to its relatively low case burden compared to other endemic areas [1,2]. While countries such as El Salvador and Belize have recently been certified malaria-free [1,2], others—including Panama, located at the southernmost end of Mesoamerica—continue to face major transmission challenges.

**Figure 1:**
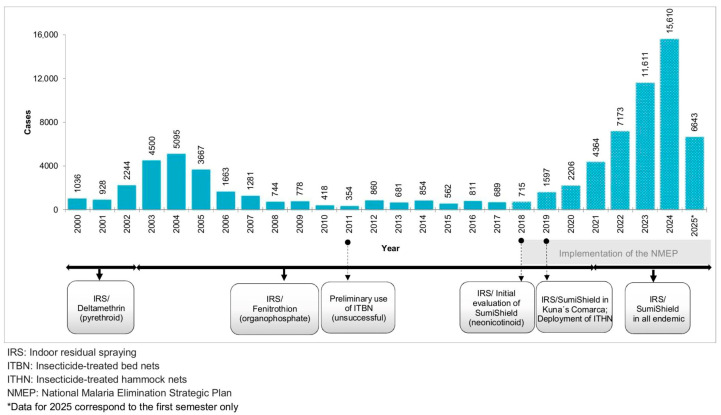
Annual malaria cases in Panama from 2000 to 2025 (first semester) highlighting major events related to vector control interventions applied in the country since 2000.

In Panama, local malaria cases rose sharply from 375 in 2015 to over 15,600 in 2024, representing an increase of more than 4000% (Figure 1). Much of this transmission occurs in indigenous comarcas and along migration routes in the Darién region [3]. Factors contributing to this resurgence include climate change, large-scale human migration, and growing concerns over the reduced effectiveness of interventions, partly driven by emerging insecticide and drug resistance [4,5].

Vector control remains a cornerstone of malaria prevention in Panama, relying primarily on indoor residual spraying (IRS) and, more recently, insecticide-treated nets (ITNs) [6,7]. Since the establishment of the National Malaria Control Program in 1957, multiple classes of insecticides (organochlorines, organophosphates, carbamates, pyrethroids, and neonicotinoids) have been extensively used (Figure 1), often selected based on availability or international recommendations rather than local surveillance data. Despite decades of intensive insecticide use, there is very limited information on the status and distribution of insecticide resistance in *Anopheles albimanus*, the most abundant and widespread malaria vector in Panama [8,9].

Addressing this gap is critical because resistance threatens the efficacy of vector control interventions and the country’s ability to meet elimination targets. In this study, we investigated the molecular resistance profile of *An. albimanus* populations in Panama by detecting the presence, frequency, and distribution of resistance alleles in three key genes: *ace-1* (acetylcholinesterase), *vgsc* (voltage-gated sodium channel), and *rdl* (GABA receptor). We also assessed the *Plasmodium* infection status of collected mosquitoes and conducted a preliminary analysis of population genetic diversity to explore potential geographic structuring.

## 2. Materials and Methods

### 2.1. Study Area

**Figure 2:**
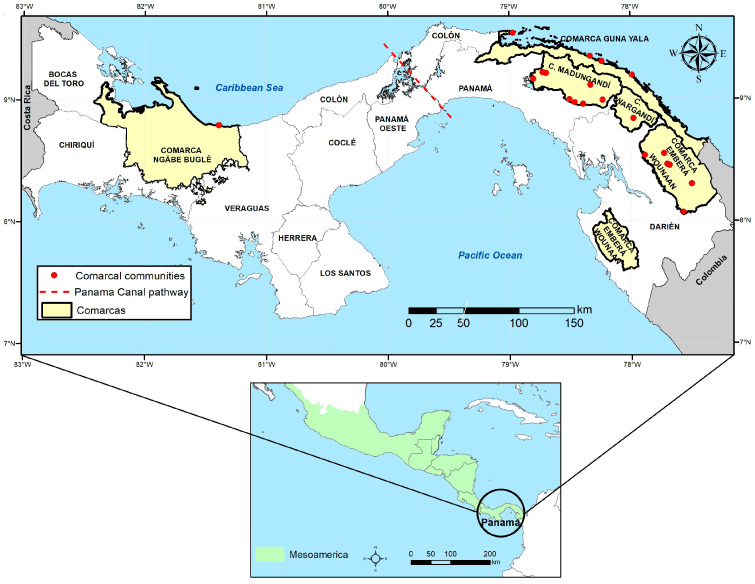
Geographic distribution of *Anopheles albimanus* sampling sites in Panama. Red dots indicate the locations of sampling sites within the indigenous regions (“Comarcas”), which are highlighted in yellow. The lower inset map shows the location of Panama within the Mesoamerican region, highlighted in green. Red dashed lines represent the Panama Canal, which separates the country into Eastern and Western regions.

Panama is located in the southernmost part of the Mesoamerican subregion, which includes southeastern Mexico and all Central American nations (Figure 2). The country is bordered by Costa Rica to the west and Colombia to the east and is flanked by the Caribbean Sea to the north and the Pacific Ocean to the south, forming a biological corridor that joins South America with the rest of Central America. The climate and natural vegetation of most parts of Panamá are typically tropical rainforest, highly suitable for breeding of malaria vectors. The country has a bimodal climate season, with a dry season characterized by an almost total absence of rain from January to March. The rainy season spans from May to November. April and December are considered transition months. Humidity is high throughout the year in most of the country, but during the rainy season, it can reach almost 100% [10].

The Panama Canal pathway has artificially divided the country into Eastern and Western Panama. Administratively, the country is organized into ten provinces and five indigenous semi-autonomous regions called “comarcas” (Figure 2). Four of these comarcas are located East of the Panama Canal (Guna Yala, Madungandí, Wargandí and Emberá Wounáan), and one West of the Panama Canal (Ngäbe Buglé). Around 17.0% of the estimated population of the country (4,064,780 inhabitants by 2023) lives within these indigenous territories that occupy 22.2% of the country’s area [11]. During the past few decades, malaria transmission in Panama has clustered across these comarcas, representing about 90 percent of all diagnosed cases in the country [12].

### 2.2. Mosquito Collections and Identification

Populations of *An. albimanus* collected between 2011 and 2023 were retrospectively analyzed. Mosquitoes were collected by human-landing catches from the peridomicile of historically endemic localities under epidemiological surveillance across the five indigenous comarcas in Panama (Figure 2). Adult mosquitoes were morphologically identified using keys for *Anopheles* [13]

### 2.3. Ethical Statement

The retrospective molecular analysis of the mosquito samples for this study was granted exemption from the Comité Institucional para el Buen Uso y Cuidado de los Animales (CIUCAL) del Instituto Conmemorativo Gorgas de Estudios de la Salud (No. 005/CIUCAL/ICGES-2024).

### 2.4. Genomic DNA Extraction and Plasmodium Infection

Samples were processed in ‘pools’ containing five *An. albimanus* per pool. Genomic DNA from these pools was isolated using the commercial DNeasy Blood and Tissue Kits (Qiagen, Hilden, Germany), following the manufacturer’s protocol. Two molecular methods were performed to evaluate the *Plasmodium* infections in the mosquito samples. Samples collected between 2011 and 2018 were analyzed using a nested PCR targeting the small subunit ribosomal RNA (ssrRNA) genes following a modified methodology from the protocol by Snounou et al. [14]. Specifically, as *P. vivax* and *P. falciparum* are the only malaria species circulating in Panama, the second PCR reaction included species-specific primers for these two parasites exclusively. The remaining *An. albimanus* pools collected between 2021 and 2023 were also analyzed by a qRT-PCR protocol targeting mitochondrial COX I and COX III genes as previously described [15].

### 2.5. Amplifications and Sequencing of Insecticide Resistance Genes

#### 2.5.1. *Vgsc* Gene

A 225 bp fragment of the *kdr* region in the *vgsc* gene was amplified using primers designed for *An. albimanus*, following protocols proposed by Lol et al. (2019) [16]: AAKDRR: (5′-GCAANGCTAAGAANAGRTTNAG-′3), AAKDRF: (5′-AGATGGAAYTTYACNGAYTTC-′3), and AAKDRF2: (5′-CATTCATTTATGATTGTGTTTCGTG-′3). The PCR mixture (25 μL) consisted of 7.7 μL of water, 12.5 μL of PCR Master Mix, 2x (Promega, Madison, WI, USA), 1 μL of MgCl_2_ (25x), 0.4 μL of each primer (AAKDRR and AAKDRF), and 5 μL of DNA template. The primary PCR conditions were set to 95 °C for 3 min, followed by 35 cycles of 95 °C for 45 s, 40.5 °C for 45 s, and 72 °C for 1 min with a final extension at 72 °C for 5 min in a SimpliAmp Termocycler (Applied Biosystems, Waltham, MA, USA). A second PCR was performed using the AAKDRR and AAKDRF2 primers with the same reaction specifications as in the primary PCR and the product of the first PCR as template DNA. The second PCR conditions consisted of 95 °C for 3 min, followed by 40 cycles of 95 °C for 45 s, 51.5 °C for 45 s, and 72 °C for 1 min with a final extension at 72 °C for 5 min in a SimpliAmp Termocycler (Applied Biosystems, Waltham, MA, USA).

#### 2.5.2. *ace-1* Gene

For the *ace-1* gene, specific primers for *An. albimanus* were used to amplify a 193 bp fragment that flanks the target codon position 119 (AAace1F 5′-TGTGGAACCCAAATAC GC’3 and AAace1R 5′-ACGTTCTCTTCCGAGGCG’3). The PCR reaction with a final volume of 25 μL consisted of 5.5 μL of water, 12.5 μL of PCR Master Mix, 2x (Promega, Madison, WI, USA), 1 μL of MgCl_2_ (25x), 0.5 μL of each primer (AAace1F and AAace1R), and 5 μL of DNA template. The cycle conditions were 94 °C for 5 min, followed by 30 cycles of 94 °C for 30 s, 60 °C for 30 s, and 72 °C for 60 s, and a final extension at 72 °C for 5 min [17].

#### 2.5.3. *Rdl* Gene

Initial attempts to amplify a fragment of the *rdl* gene of various anopheles species from Indonesia following the protocol described by Asih et al. (2012) were unsuccessful [18]. Therefore, we designed a set of specific primers for *An. albimanus* based on published sequence of the *rdl* gene from this species (NCBI RefSeq accessions: NC_050157.1 and XM_035935106.1). The primers’ specificity of the primers, as well as the identification of secondary structures and potential dimerization were assessed using the OligoAnalyzer tool (OligoAnalyzer Tool—Primer analysis and Tm Calculator|IDT, Coralville, IA, USA, https://www.idtdna.com/pages/tools/oligoanalyzer, accessed on 15 April 2024).

The designed primers—Fwd1 (5′AGTATAGCTGTGTAAGAGTC-3′) and Rev2 (5′-AGCAAATACGGAACTGAACC-3′)—were successfully used to amplify a 507 bp fragment encompassing the target codon position at codon 302 from *An. albimanus*. Amplification reactions were performed in a final volume of 25 μL containing 12.5 μL of PCR Master Mix, 2x (Promega, Madison, WI, USA), 0.4 μL of each primer (Fwd1 and Rev2), and 3 μL of DNA purified from *An. albimanus* pools. The following PCR cycling conditions were used: 94 °C for 3 min; 35 cycles of 95 °C for 30 s, 50.5 °C for 30 s, 72 °C for 1 min, and a final extension at 72 °C for 5 min.

PCR products were confirmed via electrophoresis in a 2% agarose gel with a 0.5X TBE buffer stained with SYBR™ Safe DNA Gel Stain (Invitrogen, Waltham, MA, USA). The amplicons were then purified using the QIAquick purification kit (Qiagen, Hilden, Germany). To ensure suitability for sequencing, the quality and concentration of the DNA were evaluated by measuring the absorbance at 260 and 280 nm with a NanoDrop spectrophotometer (Thermo Scientific, Waltham, MA, USA). Purified PCR products were then directly sequenced in both directions with the Sanger method using the same primers for each gene (*vgsc*, *ace-1*, and *rdl*) described for amplification and an ABI Prism 3500 XL130 sequencer (Applied Biosystems, Foster City, CA, USA). Chromatograms were visually checked, and nucleotide or amino acid consensus sequences were edited and aligned using Sequencher v4.1.4 [19] and Molecular Evolutionary Genetics Analysis (MEGA) v12.0 software (Pennsylvania State University, Center, PA, USA) [20]. Identity verification of the sequences obtained was performed by comparison with *vgsc*, *ace-1*, and *rdl* gene sequences available in GenBank, using the NCBI Blast tool (https://blast.ncbi.nlm.nih.gov/Blast.cgi, accessed on 1 June 2024). Nucleotide sequences from this study were submitted and registered in GenBank (Table S1).

### 2.6. Phylogenetic and Genetic Diversity Analyses

To explore genetic diversity and conduct a preliminary population structure analysis among *An. albimanus* populations from various endemic regions, 30 representative sequences of the three insecticide resistance genes (*vgsc*, *ace-1*, and *rdl*) were concatenated into a single sequence for each sample of 629 bp. The selection of this subset of sequences was based on strict quality criteria to ensure data reliability and comparability across loci. Only samples that yielded full-length, high-quality Sanger sequences for all three genes (*vgsc*, *ace-1*, and *rdl*) were included in the concatenated dataset.

Multilocus sequences were aligned using MEGA 12.0 software, and a phylogenetic tree was constructed by the maximum likelihood method with the Tamura model and 1000 bootstrap replicates. To create a haplotype network, PopART program version 1.7 and TCS algorithm were employed [21], utilizing the 30 concatenated samples from the three genes.

To compute genetic diversity indices, samples were grouped by their geographic origin into Western Panama and Eastern Panama. DnaSP Version v6 based on individual genes was used to estimate the number of haplotypes (H), haplotype diversity (Hd), segregating sites (S), nucleotide diversity (π), total number of mutations (Eta), the mean number of pairwise differences (k), and neutrality tests (Fu’s Fs statistics) [22].

### 2.7. Data Analysis

The data collected was registered in Microsoft Excel. The allelic frequencies from mutations detected in the three resistance genes were calculated using R 2023 (version 4.3.2, Build 513, Posit Software, PBC, Boston, MA, USA) [23].

## 3. Results

Molecular screening was performed in 891 adult female *An. albimanus* specimens (collected between 2011 and 2023) distributed in 162 pools: 120 pools from Madungandí comarca, 7 from Guna Yala, 2 from Wargandí, 22 from Emberá Wounaan and 11 from Ngäbe Buglé. Pools were molecularly examined to detect natural infection with *Plasmodium* and were sequenced to assess mutations in genes (*vgsc*, *ace-1* and *rdl*) associated with resistance to commonly used insecticides for malaria vector control. All reported allele frequencies are based on pooled samples and therefore represent pool-level detection rather than individual genotypes or zygosity.

### 3.1. Plasmodium Infection in Collected An. albimanus

A total of 680 mosquito pools were initially screened for *Plasmodium* infection, of which 47 were positive (6.9%). Pool sizes ranged from 1 to 10 mosquitoes, yielding an estimated pooled prevalence of 0.043 (95% CI: 0.036–0.050). For the present analysis, we included a subset of 162 pools, selected primarily based on the availability of high-quality DNA and *Plasmodium* positivity to allow for downstream molecular and genetic analyses. Consequently, 100 of these 162 pools tested positive for *Plasmodium*. Of the 100 positive pools, 84 were found positive for *P. vivax* (85.81%), 9 pools for *P. falciparum* and 7 were mixed infections of both species (5.1%). *Plasmodium falciparum* infections were detected in pools from *Anopheles* collected in the Madungandí comarca between 2020 and 2023 (Table S2).

Because this subset was not randomly selected and was intentionally enriched for positive samples, the proportion of positive pools in this dataset does not represent the actual prevalence of *Plasmodium* infection in the field populations but rather reflects the targeted design of this molecular analysis.

### 3.2. Frequency and Distribution of vgsc Genotypes

Of the 162 *An. albimanus* pools, 122 sequences corresponding to segment 6 of domain II of the *vgsc* gene were successfully genotyped. All sequences were aligned and compared with reference *vgsc* gene sequences from *An. albimanus* available in GenBank under accession numbers OL630652.1, KF137581.1, and MN087506.1. The wild-type allele (susceptible to pyrethroid) at codon 1014 (TTG; leucine, L1014) was present in 64 sequences (52.5%), while the wild-type allele at codon 973 (CAT; histidine, H973) was found in 28 sequences (22.9%).

**Figure 3:**
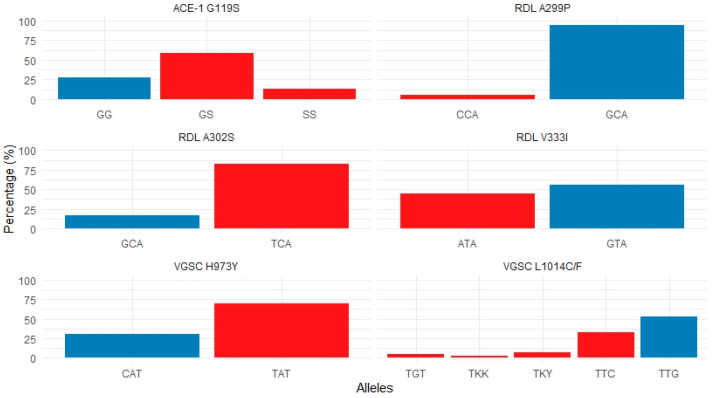
Frequencies of insecticide resistance and susceptible alleles in *Anopheles albimanus* populations from Panamanian comarcas. Red bars indicate alleles associated with insecticide resistance, while blue bars indicate susceptible alleles. The analyzed genes and their detected variants are: *vgsc* (L1014C/F and H973Y; associated with pyrethroid resistance), *ace-1* (G119S; associated with carbamate and organophosphate resistance) and *rdl* (A299P, A302S and V333I; associated with dieldrin and cyclodiene resistance). Allele frequencies were calculated from pooled mosquito samples, with each pool treated as a single observation rather than individual mosquitoes.

Conversely, pyrethroid resistance-associated mutations resulting in amino acid substitutions were identified at both codons (1014 and 973). At codon 1014, the well-characterized knockdown resistance (*kdr*) mutation was detected in almost half of the sequences, including TTC (phenylalanine, L1014F) in 6 sequences (4.9%) and TGT (cysteine, L1014C) in 40 sequences (32.8%). Additionally, heterozygous alleles were identified at this codon: TKY in 9 sequences (7.3%) and TKK in 3 sequences (2.5%); all originating from pools collected in the eastern comarcas of Madungandí and Emberá-Wounaan (Figure 3 and Figure S1). The *kdr* mutation was found to be fixed in *An. albimanus* pools analyzed from eastern regions since 2021 (Table S3).

At codon 973, the mutation TAT (tyrosine, H973Y), also implicated in resistance to pyrethroids, was observed in 64 sequences (52.5%); all of which were homozygotes and originated from eastern comarcas. Overall, 50.8% of the *kdr*-associated mutant alleles (L1014F/C and H973Y) were detected in pools from communities within the Madungandí Comarca (n = 62), located east of the Panama Canal (Figure 3 and Figure S1). In contrast, none of the *vgsc* sequences analyzed in this study from the Ngäbe-Buglé comarca located west of the Panama Canal, exhibited mutation associated with pyrethroids resistance (Table S3).

### 3.3. Frequency and Distribution of ace-1 Genotypes

All *ace-1* sequences from this study were aligned with reference acetylcholinesterase-1 gene sequences from *An. albimanus* (GenBank accessions AJ566403.1 and AJ566402.1) and *An. darlingi* (MK477204.1). All three possible genotypes of the *ace-1* gene were identified at codon 119 in 132 sequences analyzed. These included the homozygous susceptible genotype (119GG) found in 36 sequences (27%), the heterozygous genotype (119GS) in 77 sequences (58%) and the homozygous resistant genotype (119SS) found in 18 samples (14.7%), which is associated with resistance to organophosphorus (OP) and carbamate (CM) insecticides. The resistance-associated mutation (G119S) was detected in samples from communities in eastern Panama, including Madungandí (n = 15), Emberá-Wounaan (n = 2), and Wargandí (n = 1) (Figure 3 and Figure S1 and Table S3). Notably, the G119S mutation was absent in samples collected from western Panama.

### 3.4. Frequency and Distribution of rdl Genotypes

Three non-synonymous mutations were identified in the *rdl* gene based on the analysis of 91 *An. albimanus* sequences from this study. All sequences were aligned with the reference *rdl* gene sequence from *An. funestus*, available in GenBank under accession number MN562790.1. Nucleotide variations resulting in corresponding amino acid changes were observed at codons 299, 302, and 333 (Table S3). All identified alleles in the *rdl* gene from this study were found in homozygous form.

At codon 299, the wild-type allele GCA, which encodes alanine (A299), was found in 86 sequences (94.5%). A mutant variant encoding proline (A299P; CCA) was detected in five sequences (5.5%), including four from eastern comarcas and one from the Ngäbe-Buglé Comarca (n = 1) in western Panama (Figure 3 and Figure S1 and Table S3).

At codon 302, the well-characterized resistance-associated mutation (A302S) that results in the substitution of the amino acid alanine (GSA) with serine (TCA), was detected in 67 sequences (73.6%) (Figure 3). This A302S substitution was found predominantly in samples from eastern Panama, including Madungandí (n = 56), Emberá-Wounaan (n = 11) and Wargandí (n = 3), as well as in one sample from the western comarca of Ngäbe-Buglé (n = 1) (Figure S1).

An additional polymorphism was detected at codon 333. The wild-type allele GTA, which encodes valine (V333), was identified in 45 sequences (49.5%), while a variant allele ATA (V333I) encoding isoleucine was found in 36 sequences (39.5%). This V333I variant was observed in *An. albimanus* pools from communities in Madungandí (n = 29) and Emberá-Wounaan (n = 6) in eastern Panama, and in one sample from the Ngäbe-Buglé Comarca in western Panama (Figure 3 and Figure S1 and Table S3).

**Figure 4:**
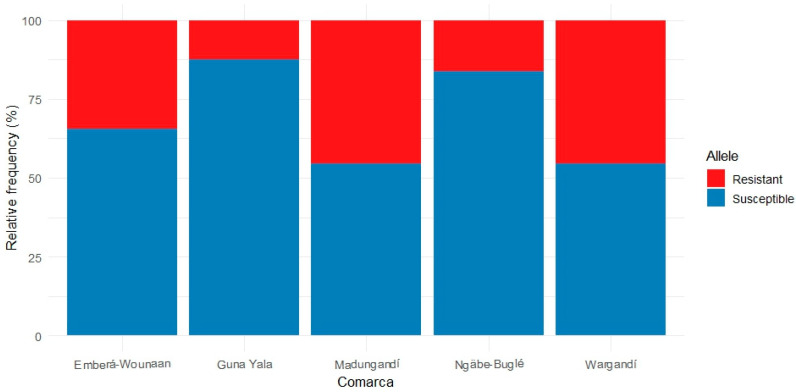
Relative frequency (%) of resistant and susceptible alleles for *vgsc*, *ace*-1 and *rdl* genes across the different comarcas. Resistant alleles (red) predominate in some comarcas, suggesting a higher potential for insecticide resistance to pyrethroids (*vgsc*), organophosphates/carbamates (*ace*-1), and cyclodienes and dieldrin (*rdl*), while other comarcas exhibit a greater proportion of susceptible alleles (blue), indicating lower prevalence of resistance-associated variants. Allele frequencies were calculated from pooled mosquito samples, with each pool treated as a single observation rather than individual mosquitoes.

The total number of alleles (resistant and susceptible) detected in the *An. albimanus* pools from each of the five comarcas studied is summarized in Figure 4. Although there was considerable variability in the number of samples analyzed from each comarca, Madungandí exhibited the highest number of detected alleles, followed by Wargandí and Emberá-Wounaan. In contrast, the eastern comarcas of Guna Yala and Ngäbe-Buglé in the west, displayed lower frequencies. The ratio of resistant to susceptible alleles also varies among comarcas, suggesting regional differences in genetic resistance patterns.

### 3.5. Phylogenetic and Genetic Diversity Results

**Figure 5:**
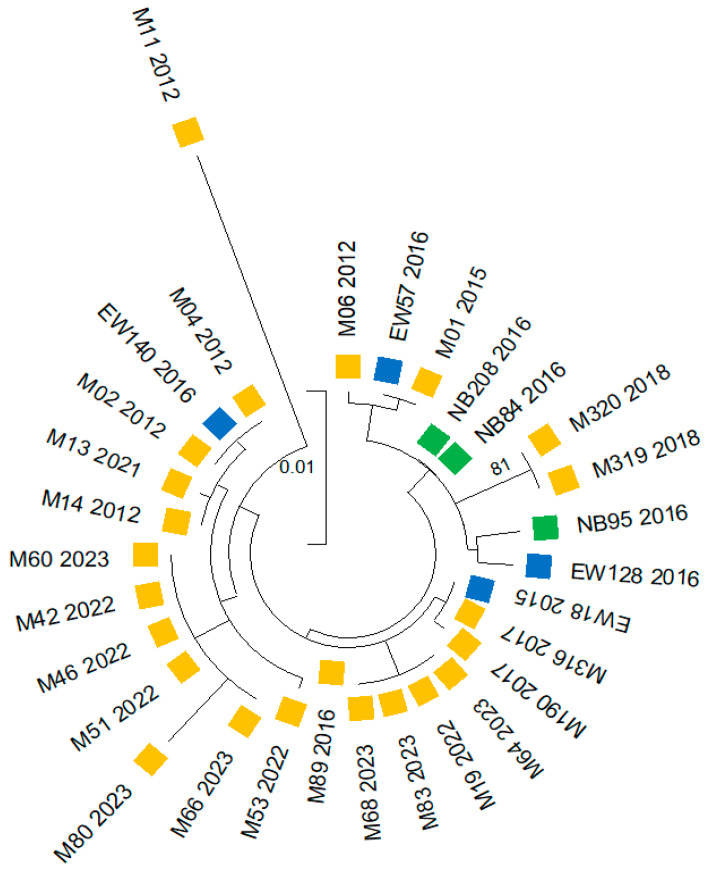
Phylogenetic tree based on concatenated sequences of three insecticide resistance genes (*vgsc*, *ace*-1, and *rdl*) from 30 pools of *Anopheles albimanus* collected between 2011 and 2023 across three comarcas in Panama: Madungandí (n = 23, yellow), Emberá-Wounaan (n = 4, blue), and Ngäbe-Buglé (n = 3, green). The tree was inferred using the Maximum Likelihood method under the Tamura 3-parameter model. The final dataset comprised 30 concatenated nucleotide sequences with a total aligned length of 629 bp. Because these loci are associated with insecticide resistance, they may be subject to selection and thus do not necessarily represent neutral population structure.

Phylogenetic tree reconstruction and a haplotype network analysis were conducted using concatenated sequences of the three insecticide resistance markers (*vgsc*, *ace-1* and *rdl* genes) from *An. albimanus* collected between 2011 and 2023 across three comarcas (Madungandí and Emberá-Wounaan from the east, and Ngäbe-Buglé from the west). The phylogenetic tree was inferred using the Maximum Likelihood method and Tamura model. The final analysis incorporated 30 nucleotide sequences, covering 629 positions in the final dataset (Figure 5 and Table S1). The complete sequence alignment of the concatenated genes from this study is available upon request.

With the exception of a single sample (M11 2012), all samples grouped in a predominant, well-defined clade that encompassed samples from the three comarcas and from the multiple collection years, indicating the absence of a dominant genotype circulating, and instead suggests the persistence of diverse genotypes coexisting over time (Figure 5). Within this predominant clade, several subclades were observed, reflecting a greater genetic diversity among samples generated from eastern comarcas mainly from Madungandí, which clustered into distinct subclades with closer genetic relatedness between them. In contrast, sequences from the Ngäbe-Buglé comarca located in western Panama formed a separate subclade together with some eastern samples from Madungandí and Emberá-Wounaan, indicating some degree of genetic connectivity.

The distinct sample M11 2012 was collected from Puente Bayano, a hyperendemic community located in the eastern comarca of Madugandí. This sample was grouped apart in a different clade, highlighting its genetic distance from the rest of the samples analyzed.

**Figure 6:**
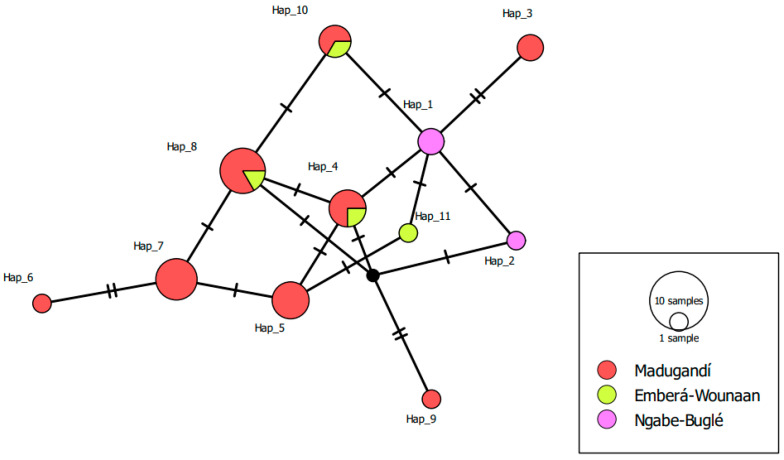
Haplotype network constructed using the median-joining algorithm implemented in the TCS method, based on concatenated sequences of three insecticide resistance genes (*vgsc*, *ace*-1, and *rdl*) from 30 pools of *Anopheles albimanus* collected between 2011 and 2023 across three comarcas in Panama. Each circle represents a unique haplotype, with colors indicating geographic origin and circle size proportional to haplotype frequency. The lines connecting haplotypes indicate mutational steps, and small crossbars along the lines correspond to the number of nucleotide substitutions. These loci are known targets of insecticide selection pressure and therefore may not accurately reflect neutral population genetic structure.

The haplotype network analysis, based on the concatenated gene sequences revealed the presence of 11 distinct haplotypes (Figure 6). Most haplotypes were restricted to a single comarca, indicating localized circulations of specific genetic variants. For example, haplotypes 3, 5, 6, 7 and 9 were only found in Madugandí, while haplotype 11 was restricted to the comarca Emberá-Wounaan, both located in eastern Panama. In contrast, haplotypes 1 y 2 were exclusively identified in the comarca Ngäbe-bugle in western Panama. Haplotypes 4, 8 and 10 contained samples from different eastern comarcas and showed genetic relatedness between them, indicating potential gene flow or shared ancestry among populations in that region.

To explore potential geographic structuring and population differentiation among *An. albimanus* populations collected in this study, a preliminary analysis of the genetic diversity was performed. For this purpose, samples were grouped according to the collection site into two ecologically and geographically distinct malaria-endemic regions of Panama (Eastern and Western). Genetic diversity indexes were calculated and compared for each of the three resistance-associated genes (*vgsc*, *ace-1* and *rdl*) (Table S4). Higher diversity indices were captured with the *vgsc* gene (*kdr*) and was therefore chosen for further diversity analysis.

Substantial differences in genetic diversity were observed among both groups. Eastern populations exhibited a high genetic diversity, with 7 segregating sites, 14 haplotypes, and a haplotype diversity of 0.77. The nucleotide diversity (ᴫ = 0.078) and the presence of 8 different mutations, reflect the heterogeneous origins of these specimens. The negative Fu’s Fs statistic (−5.89) is consistent with recent population expansion or that *Anopheles* populations from this region are under selective pressure.

In contrast, western Panama populations displayed low genetic diversity, with only two haplotypes identified. Most specimens were genetically similar (Hd = 0.48; ᴫ = 0). This homogeneity suggests a genetic bottleneck, possibly due to limited gene flow, localized vector control pressure or ecological constraints.

## 4. Discussion

After falling short of meeting the 2025 malaria elimination goals set by the WHO Global Technical Strategy (GTS), the NMEP in Panama is currently adjusting strategies to address issues that might have hindered elimination progress. A key component of this revised strategy needs to include a more effective and evidence-based vector control program, guided by local epidemiological and entomological data, including a program for monitoring and managing insecticide resistance [24]. In this context, this study provides the first molecular evidence in the country on how allelic variants in *vgsc*, *ace*-1, and *rdl* genes might be contributing to the resistance status to insecticides in *An. albimanus* populations, the dominant malaria vector in Panama across malaria endemic comarcas.

Over the past several decades, malaria transmission in Panama has remained disproportionally clustered in indigenous comarcas that together occupy approximately 22.0% of the Panamanian territory [25]. Indeed, malaria cases in these reservations have accounted for more than 90% of all cases diagnosed in the country in recent decades [12]. Indigenous communities living in comarcas are highly underserved, with substantially higher rates of extreme poverty and limited healthcare compared to the rest of the country [12,25]. These areas are not generally exposed to the high amounts of agrochemicals used in large-scale crop plantations, since their indigenous inhabitants relied mainly on subsistence agriculture and fishing. However, these areas have been subjected to recurrent applications of multiple classes of insecticides used primarily as IRS by vector control programs. This continued exposure might have exerted strong selection pressure over vector populations, therefore favoring the emergence of resistance alleles.

At the initial phases of NMP in Panama (from the early 1960s to the 1980s), organochlorides (DDT and dieldrin) and carbamates (Propoxur) were widely used as part of the IRS vector control program [12]. Over the past 25 years, these insecticides have been replaced by chemicals from the pyrethroid, organophosphate, and neonicotinoid classes. Pyrethroids (Deltamethrin) were used between 1996 and 2002, followed by the organophosphate fenitrothion (Sumithion) from 2002 to 2020 (Figure 1). In September 2019, a neonicotinoid insecticide (Sumishield) was introduced as an alternative to fenitrothion in three eastern highly endemic comarcas: Madungandí, Guna Yala and Wargandí. Its use was later expanded to the remaining malaria-endemic regions beginning in 2021 (Figure 1) [9]. However, in most cases, changes in insecticides for IRS have been driven primarily by product availability or international policy recommendations rather than by thorough local evaluations of insecticide effectiveness and resistance status of the local vectors.

To evaluate the molecular resistance status of *An. albimanus* in Panama, we analyzed mutations in three genes (*vgsc*, *ace*-1, and *rdl*) shown to be strongly associated with resistance to common insecticides. *vgsc* gene is the primary target of pyrethroids and DDT (dichlorodiphenyltrichloroethane), and mutations at codons 973 and 1014 have been shown to confer resistance to both insecticides in *Anopheles* mosquitoes [26,27,28]. In this study, both mutations (H973Y and L1014F/C) were detected at high frequencies (50.8%) in eastern comarcas but were absent in comarcas located west of the Panama Canal (Figure 3 and Table S3).

Acetylcholinesterase is the primary molecular target of organophosphates and carbamates, and genetic variants in this gene have been clearly recognized to confer resistance to both insecticides in *Anopheles* populations [28,29]. The resistance-associated mutation (G119S) was widespread in eastern comarcas (over 70% of the samples evaluated) but was not detected in western comarcas (Figure 3 and Table S3).

Three non-synonymous mutations (A299P, A302S, V333I) were identified in the *rdl* gene, the primary target for insecticides of several insecticides, including cyclodienes (dieldrin) (Figure 3 and Table S3). This gene has also been identified as a potential secondary target for other insecticide classes, including neonicotinoids and pyrethroids [30]. The resistance-related amino acid substitution A302S was detected at high frequencies (73.6%) across all studied comarcas, particularly in the east. A valine to isoleucine amino acid substitution was detected at codon 333 (V333I) in 39.5% of the samples. A similar amino acid replacement has been described at codon 327 in *Anopheles* species from Africa and Asia [31,32]. Further studies are needed to assess the impact of the V302I mutation in *An. albimanus* populations. An additional non-synonymous substitution was detected at low frequencies (5.5%) at codon 299 (alanine to proline) on the *rdl* gene (A299P). The biological implication of this amino acid substitution in *An. albimanus* populations warrants further investigation.

Dieldrin or other cyclodienes have not been used in the country by the NMP since the early 1960s. Therefore, the existence of *rdl* mutant alleles in *An. albimanus* populations could be associated with the use of other insecticides by NMP that also target *rdl*, such as neonicotinoids. It is also possible that these mutant alleles are stable in the absence of insecticide pressure or that they confer a selective advantage compared to the wild types.

Few studies have evaluated the presence and frequency of insecticide resistance-associated mutations in dominant *Anopheles* populations from Mesoamerica (from Panama to southern Mexico). Resistant *kdr* alleles have been previously reported in *An. albimanus* populations from México, Nicaragua and Costa Rica [33,34]. Conversely, a recent study did not detect *kdr* or *ace*-1 mutations in *An. albimanus* from the State of Quintana Roo in southeastern Mexico [35]. This is the first report of *vgsc*, *ace*-1, and *rdl* resistance-associated mutations in *An. albimanus* populations from Panama.

Our molecular findings in Panama complement recent research in neighboring Central American countries. In a comparative study of *An. albimanus* populations from Peru and Guatemala, Mackenzie-Impoinvil et al. (2019) reported no target-site resistance mutations in the *vgsc* and *ace*-1 genes from Guatemalan samples, while *rdl*-associated mutations were detected at low frequencies [36]. Furthermore, a recent study by Escobar et al. (2022) provided updated information on the distribution, diversity and abundance of *Anopheles* species in malaria-endemic and non-endemic areas of Honduras, highlighting ecological differences that may influence vector competence and exposure to insecticides [37]. Integrating this regional data contributes to a broader understanding of the spatial and ecological dynamics that may influence insecticide resistance in malaria vector populations from Central America.

A higher molecular infection rate by *P. vivax* was observed in the *An. albimanus* pools collected from all comarcas (Table S2), a finding consistent with the high *P. vivax* transmission reported in humans during the collection years. Notably, *P. falciparum* infections were detected in pools collected in recent years (2022–2023) from communities within the eastern comarca of Madungandí (Table S3). This finding preceded the official confirmation of human *P. falciparum* cases in 2024 from the same communities where infected vectors were found. Autochthonous *P. falciparum* transmission was virtually eliminated in the country, but it has now re-emerged in the eastern comarcas of the country. In this context, monitoring infection in mosquito vectors for *Plasmodium* species can be helpful as an important early warning tool to anticipate changes in human malaria transmission, including the possible resurgence of *Plasmodium* species in a specific area.

Although insecticide-resistance genes may not be ideal for conducting genetic diversity studies in *Anopheles* due to the strong selective pressures to which they are frequently subjected, our results evidence a distinct *An. albimanus* population structure between eastern and western comarcas (Table S4). Mosquitoes from the western side were highly homogenous, suggesting reduced genetic diversity or evidence of a genetic bottleneck, possibly due to limited gene flow or ecological isolation. In contrast, eastern samples exhibited a much higher genetic diversity, possibly influenced by geographical, ecological and epidemiological differences that characterize this region that includes a higher vector species abundance/diversity and intense malaria transmission rate compared to the western side. Although samples were grouped broadly into eastern and western Panama for analysis, this strategy may mask finer-scale structure. Given the uneven representation of comarcas, east–west comparisons must be interpreted with caution and considered exploratory.

Our study has several important limitations. First, the number of pools analyzed was relatively small and with a limited timeframe, spanning from 2011 to 2023. There was also an uneven distribution of the number of pools collected across the comarcas, with a considerable underrepresentation of samples from the western comarca of Ngäbe Buglé. Consequently, our results may not necessarily capture the entire or current insecticide-resistance profile or the diversity of *An. albimanus* populations in the country. However, it is noteworthy to mention that collecting mosquitoes in malaria-endemic regions in Panama is logistically challenging. Most of these areas are remote and difficult to reach, particularly western comarcas during the rainy season. Transporting mosquito samples from remote areas to specialized laboratories for molecular testing is also time-consuming and costly. Another limitation of our study is that the amplicons analyzed for *vgsc* and *rdl* were relatively short. While sufficient to detect well-characterized resistance-associated variants, these short fragments may not capture the full spectrum of possible mutations across the genes, and thus, the actual diversity of resistance alleles could be underestimated.

Analyzing *Anopheles* mosquitoes in pools instead of individually is also major limitation that can not only negatively impact an accurate estimation of allele frequencies but also affect the estimation of population genetic parameters, such as determining allele zygosity of the mutations associated with insecticide resistance. Nevertheless, pool analysis can be an efficient and cost-effective approach when working in most malaria-endemic settings, characterized by resource constraints. It is also an efficient strategy when the goal is rapid screening of common mutations or a preliminary assessment to identify population structure.

Despite the limited and unbalanced sample size across the comarcas, our study provides a valuable baseline for planning future molecular vector surveillance studies in the region. It also provides valuable information to guide insecticide selection for IRS performed by the NMEP in Panamá. Nevertheless, our molecular results for assessing insecticide resistance in *Anopheles* should be confirmed with additional susceptibility phenotypic bioassays, such as the WHO tube test and CDC bottle assay.

In conclusion, resistance-related mutations to all four main classes of insecticides were present at high frequencies in *An. albimanus* populations from eastern Panama, while they were absent or at low frequencies in populations from the western region. These findings emphasize the need to implement region-specific vector control strategies.

## Data Availability

All data underlying the results from this study are provided as part of the article in tables and figures. DNA sequences were deposited in GenBank as described in the methodology.

## Supplementary Materials

The following supporting information can be downloaded at https://www.mdpi.com/article/10.3390/insects16111115/s1. Figure S1: Frequency of resistance-related alleles in *vgsc*, *ace*-1 and *rdl* genes at each study area (comarca) by year of collection; Table S1: Genbank accession numbers and mutations detected in insecticide-resistance genes from *Anopheles albimanus* in Panama; Table S2: Prevalence of *Plasmodium* spp. in pools of *Anopheles albimanus* populations collected in the study; Table S3: Prevalence of *Plasmodium* and frequency/distribution of resistance -related alleles in *vgsc*, *ace*-1 and *rdl* genes at each study area (comarca) by year of collection; Table S4: Comparison of insecticide-resistance genes diversity among *Anopheles albimanus* collected from two geographical and ecological separate malaria-endemic regions in Panama.

## Abbreviations

The following abbreviations are used in this manuscript:

ace-1	Acetylcholinesterase 1
Hd	haplotype diversity
GABA	Gamma aminobutyric acid
IRS	Indoor residual spraying
ITN	Insecticide-Treated Nets
NMPE	National Malaria Elimination Program
PEEM	Plan for the Elimination of Malaria
rdl	Resistance to dieldrin
vgsc	Voltage-gated sodium channel
WHO	World Health Organization
