# Supplementary material for: Molecular Detection of Insecticide Resistance-Associated Mutations in vgsc, ace-1, and rdl Genes of Anopheles albimanus in Panama
Source: Insects. 2025 Oct 31;16(11):1115. doi: 10.3390/insects16111115 (PMC12653162; doi:10.3390/insects16111115)

**Figure S1**. Frequency of resistance -related alleles in *vgsc*, *ace*-1 and *rdl* genes at each study area (comarca) by year of collection


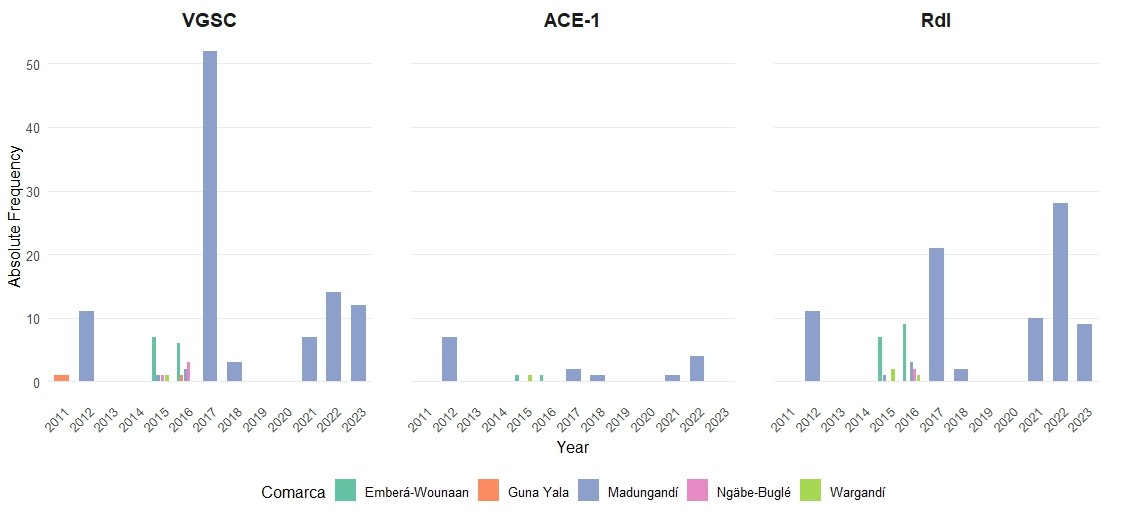

Supplement: Supplementary file 1 [file insects-16-01115-s001.zip › insects-3822769-supplementary/Figure S1 Frequency of resistance of resistance -related alleles at each study area by year of collection.docx]
