# Supplementary material for: Molecular Detection of Insecticide Resistance-Associated Mutations in vgsc, ace-1, and rdl Genes of Anopheles albimanus in Panama
Source: Insects. 2025 Oct 31;16(11):1115. doi: 10.3390/insects16111115 (PMC12653162; doi:10.3390/insects16111115)
Supplement: Supplementary file 1 [file insects-16-01115-s001.zip › insects-3822769-supplementary/Table S2 Prevalence Plasmodium pools.docx]

**Table S2**: Prevalence of *Plasmodium* spp in pools of *Anopheles albimanus* populations collected in the study

| **Study area** |  | **Pools screened** | ***Plasmodium***  **positive (%)** | ***P. vivax***  **positive (%)** | ***P. falciparum***  **positive (%)** |
| --- | --- | --- | --- | --- | --- |
| Madungandí |  | 120 | 84 (70.0) | 24 (75) | 9 (10.7) |
| Guna Yala |  | 7 | 2 (28.6) | 15 (88.2) | 0 |
| Wargandí |  | 2 | 0 | 0 | 0 |
| Emberá-Wounaan |  | 22 | 9 (40.1) | 9 (40.1) | 0 |
| Ngäbe-Buglé |  | 11 | 4 (36.4) | 4 (36.4) | 0 |
| Total |  | 162 | 100 (61.1) | 50 (78.1) | 9 (5.6) |
