# Supplementary material for: Molecular Detection of Insecticide Resistance-Associated Mutations in vgsc, ace-1, and rdl Genes of Anopheles albimanus in Panama
Source: Insects. 2025 Oct 31;16(11):1115. doi: 10.3390/insects16111115 (PMC12653162; doi:10.3390/insects16111115)
Supplement: Supplementary file 1 [file insects-16-01115-s001.zip › insects-3822769-supplementary/Table S4 Genetic diversity.docx]

**Table S5.** Comparison of insecticide-resistance genes diversity among *Anopheles albimanus* collected from two geographical and ecological separate malaria-endemic regions in Panama.

N = number of analyzed sequences; S = number of segregating sites; H = number of haplotypes; Hd = haplotypes diversity; sequences; ᴫ = Nucleotide diversity; Eta = total number of mutations; k = average number of nucleotide differences
